# Hierarchical Levels of Seed Predation Variation by Introduced Beetles on an Endemic Mediterranean Palm

**DOI:** 10.1371/journal.pone.0109867

**Published:** 2014-10-23

**Authors:** Marta Rodríguez, Miguel Delibes, José Mª. Fedriani

**Affiliations:** 1 Departament of Conservation Biology. Estación Biológica de Doñana (CSIC), Sevilla, Spain; 2 Department of Ecological Modelling. Helmholtz Centre for Environmental Research GmbH-UFZ, Leipzig, Germany; 3 Centre for Applied Ecology “Prof. Baeta Neves”, Institute Superior of Agronomy (University of Lisbon), Lisbon, Portugal; Fred Hutchinson Cancer Research Center, United States of America

## Abstract

Seed predators can limit plant recruitment and thus profoundly impinge the dynamics of plant populations, especially when diverse seed predators (e.g., native and introduced) attack particular plant populations. Surprisingly, however, we know little concerning the potential hierarchy of spatial scales (e.g., region, population, patch) and coupled ecological correlates governing variation in the overall impact that native and introduced seed predators have on plant populations. We investigated several spatial scales and ecological correlates of pre-dispersal seed predation by invasive borer beetles in *Chamaerops humilis* (Arecaceae), a charismatic endemic palm of the Mediteranean basin. To this end, we considered 13 palm populations (115 palms) within four geographical regions of the Iberian Peninsula. The observed interregional differences in percentages of seed predation by invasive beetles were not significant likely because of considerable variation among populations within regions. Among population variation in seed predation was largely related to level of human impact. In general, levels of seed predation were several folds higher in human-altered populations than in natural populations. Within populations, seed predation declined significantly with the increase in amount of persisting fruit pulp, which acted as a barrier against seed predators. Our results revealed that a native species (a palm) is affected by the introduction of related species because of the concurrent introduction of seed predators that feed on both the introduced and native palms. We also show how the impact of invasive seed predators on plants can vary across a hierarchy of levels ranging from variation among individuals within local populations to large scale regional divergences.

## Introduction

The degradation of ecosystems has altered the strength, pattern, and outcomes of many plant-animal interactions because of modifications such as habitat fragmentation, species introduction, and defaunation [1], [2], [3]. As a result, for example, the dynamics of native plant populations and even communities are altered by the combined impact of native and introduced seed predators limiting plant recruitment [4], [5], [6]. Ecological correlates of seed predation are rather species-specific and, even within a given plant species, intensity of seed predation may vary enormously depending on a myriad of factors acting at different levels [7], [8], [9], [10]. Surprisingly, however, we know little concerning the potential hierarchy of spatial scales (e.g. among regions, within population) and coupled ecological correlates governing variation in the combined effect of introduced and native seed predators on plant populations.

Insects are major seed predators in many tropical and temperate habitats [7], [11], [12], [13]. In particular, pre-dispersal seed losses by boring insects are pervasive worldwide [14], [15]. Because palms (Family Arecaceae) typically produce large seeds with nutritious endosperms [16], [17], seed boring insects often target them, lessening palm reproductive potential [17], [18], [19]. Most palm seed borers are bruchids and scolytines [20] and their significance as seed predators has been well studied in New World palms [14], [17], [18]. However, very little is known about the ecological factors determining palm seed predation in Europe, where introduced scolitines are common, especially in human-altered habitats [21], [22]. Interestingly, the only native Arecaceae in continental Europe, the Mediterranean dwarf palm (*Chamaerops humilis*), often coexists with many ornamental palm species introduced together with their respective seed predators and pests [23], [24] especially at the interface between natural and human-altered habitats ([25], Authors *unpublished data*). Identifying how introduced seed predators, in conjunction with native ones, lessen plant reproductive performance is clearly necessary to forecast the outcomes of global change in human-altered areas, such as the Mediterranean Europe [2], [26].

In this study, we investigated several levels of spatial variation in the intensity of seed predation by introduced and native beetles on *C. humilis* seeds across the Mediterranean Spain. Despite the ecological, cultural, and economic importance of *C. humilis* [19], [25], [27], we know little about the amount and ecological correlates of seed predation in natural populations. In addition, habitat degradation modifies the relative abundances of local populations and introduces new species, including ornamental palms and their seed predators (such as scolytine species; [28]). Consequently, habitat degradation can be expected to enhance *C. humilis* seed predation by non-native boring predators. On the other hand, *C. humilis* drupes are often harvested by small mammals, which feed on their fleshy pulp and leave total or partially defleshed seeds under mother plants ([19], Authors *unpublished data*). Because the flesh of many fruit species often acts as a physical and chemical barrier against invertebrate seed predators [17], [19], [29], [30], we predict that defleshed drupes, with well exposed seeds, will be more often predated by invertebrates as compared with intact drupes. Specifically, we seek to answer the following three questions: 1) How strong and variable are the interactions between introduced and native seed predators and *C. humilis* at different spatial levels (i.e. among regions, within region, and within population) in the Iberian Peninsula? 2) Does seed predation intensity vary between human-altered and natural *C. humilis* populations? and 3) Does a high amount of persisting pulp in *C. humilis* drupes lessen seed predation?

## Materials and Methods

### Study system

**Figure 1:**
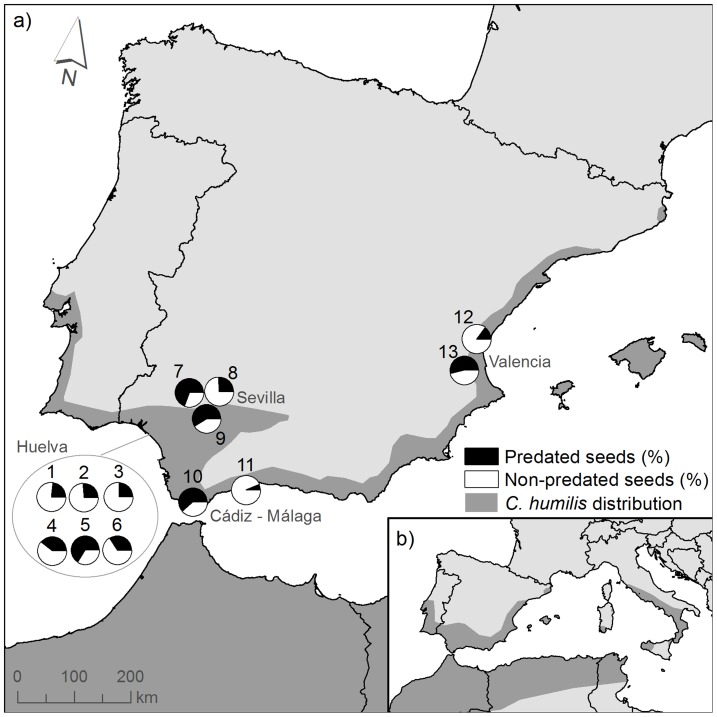
Distribution of *C. humilis* and location of sampled populations. a) Map showing the approximate distribution of *C. humilis* in the Iberian Peninsula as well as the percentages of seed predation in different regions using sector charts for each sampled population. b) Small inset showing *C. humilis* distribution in the Mediterranean basin (modified from Merlo et al. 1993). Populations: 1: Hinojos I; 2: Hinojos II; 3: Hato Ratón; 4: Matasgordas; 5: Chalet/Palacio; 6: Rocina; 7: Alamillo; 8: La Cantina; 9: Matallana; 10: Garganta Verde; 11: Fuengirola; 12: Saler; 13: Viveros.

*Chamaerops humilis* is a small dioecious palm endemic to the Western Mediterranean basin ([31]; Fig. 1). It is considered a thermomediterranean bioindicator and usually it is not present above an elevation of 1000 meters, being most common in coastal areas (Fig. 1). It is relatively abundant in Mediterranean scrub thickets and open pine forests. Due to its vigorous sprouting, *C. humilis* is very tolerant to disturbance (fire, herbivory, etc; [31]) and thus it is often used in restoration programs in arid areas. Besides, it has been used in ornamental landscaping as well as in traditional craft [27].

It flowers in March-May and is pollinated by wind and insects [31], [32]. The fruits are tridrupes grouped in bunches 5–30 cm above ground that ripen in autumn (September-November). Drupes are single-seeded, protected by a hard endocarp and a fleshy and fibrous mesocarp [19]. Since the dispersal unit is the drupe, hereafter we will refer to each drupe as fruit. Seeds are mainly dispersed by mammals such as red foxes *Vulpes vulpes* and badgers *Meles meles* [19]. Rabbits *Oryctolagus cuniculus* and some rodent species feed on the pulp of ripe and unripe fruits, generally leaving the defleshed seeds underneath mother palms ([19], Authors *unpublished data*). Defleshed seeds are often attacked by insects, with adult and larvae of introduced seed-boring beetles were observed frequently.

Beetles that bore into *C. humilis* seeds (several of them kindly identified by Dr. Alain Roques) included at least two non-native scolytine species, *Coccotrypes dactyliperda* and *Dactylotrypes longicollis* [23], [24]. *C. dactyliperda* seems to be native to the tropical areas of the Old World, but today has a circumtropical range [23]. Considered a polyphagous species, it is a regular pest of wild and ornamental palms, attacking at least 18 species [24]. When feeding on date palms *Phoenix dactylifera*, female *C*. *dactyliperda* bore the pulp of unripe fruits and the injured dates drop from the infrutescenses; later, female beetles reproduce mainly on the dropped fruits by boring the hard coat of the seeds to lay eggs inside [33]. Conversely, *D*. *longicollis* seems to be native of the Canary Islands [23]. It mainly predates Canary palm seeds [34], [35], [36], but also fallen *C. humilis* seeds with dry or deprived pulp [37].

### Sampling sites

Predation by boring insects on *C. humilis* seeds was studied in 13 populations at four geographical areas within the Iberian Peninsula (Valencia, Sevilla, Huelva, Cádiz-Málaga; Fig. 1a). Within each region, separation between adjacent populations was ≥5.5 Km. For each population, we recorded whether it was located in a natural or human-altered site, the dominant species, and other details (Table 1). Human-altered populations were those located in urban areas or very close to human settlements, usually hosting several species of ornamental palms (mainly date palm, Canary palm *Phoenix canariensis*, *C. humilis*, and *Washingtonia* spp). In all localities climate is typically Mediterranean, with two well-defined seasons: a hot dry summer from June to September and a mild rainy winter from November to February [38]. Several of our sampled Huelva populations were located within the Doñana National Park; thus, we attained permits from the authorities responsible, i.e. the Spanish National Park Service as well as the Junta de Andalucía (ref. 4225/MDCG/mect). For the remaining populations permission was not required. Our study did not involve any endangered or protected species.

**Table 1:** Summary table showing the number of *C. humilis* individuals and number of fruits sampled in each population, the habitat type, geographical region, type of population (i.e., human-altered [H] vs. natural [N]), and the percent predation and average number of holes per predated seed.

Region	Population	Coor (N; W)	Type	Habitat	#palms	#fruits	%predation	#holes
Valencia	Saler	39.39; 0.33	N	Mediterranean scrub dominated by *Pistacia lentiscus*.	7	138	15.0±6.5	2.11±0.5
	Viveros	39.48; 0.36	H	Urban park.	10	179	53.8±6.9	6.12±0.9
	**Total**				**17**	**317**		
Sevilla	Alamillo	37.42; 5.99	H	Urban park.	5	78	69.4±7.1	4.6±0.9
	La Cantina	37.64; 6.07	N	Mediterranean scrub.	3	24	25.8±14.4	1±0.0
	Matallana	37.66; 5.57	N	*C. humilis* palm grove.	9	139	59.0±8.6	3.3±0.5
	**Total**				**17**	**241**		
Cádiz-Málaga	Fuengirola	36.52; 4.64	H	Mediterranean scrub.	10	147	6.3±3.6	3.9±1.7
	Garganta verde	36.48; 5.24	N	Mediterranean scrub dominated by *Pistacia lentiscus*.	9	136	61.6±12.2	2.5±0.2
	**Total**				**24**	**360**		
Huelva	Hinojos I	37.21; 6.41	N	Mediterranean scrub dominated by *Pistacia lentiscus*.	8	101	26.2±9.0	2.0±0.3
	Hinojos II	37.26; 6.39	N	Mediterranean scrub dominated by *Pistacia lentiscus*.	10	171	23.5±11.4	3.2±1.0
	Hato Ratón	37.17; 6.33	N	Mediterranean scrub dominated by *Pistacia lentiscus*.	10	96	24.4±9.3	2.4±0.4
	Matasgordas	37.13; 6.44	N	Mediterranean scrub	10	86	39.3±10.9	2.7±0.8
	Chalet/Palacio	37.07; 6.51	H	Mediterranean scrub dominated by *Halimium halimifolium*	11	129	66.1±9.6	4.4±0.6
	Rocina	37.13; 6.52	N	Mediterranean scrub dominated by *Pistacia lentiscus*	13	138	34.4±6.9	2.9±0.5
	**Total**				**62**	**721**		

### Seed predation estimates

Seed predation was estimated from the presence and number of entrance holes made by boring insects on *C. humilis* seed endocarps. Typically, an endocarp attacked by borer beetles showed several holes, with the embryos and most endosperms consumed. These holes were located at either the distal, narrow tip of the endocarp or at the middle of endocarps. Germination pores were generally located at the proximal base and clearly differed in size and contour from holes made by borer beetles [19]. All seeds having at least one hole were classified as predated. Also, the number of holes per depredated seed was counted and used as a second estimate of the infestation level by boring insects. In each population, about ten fruiting *C. humilis* were usually considered (range: 3–13) and up to 20 fallen drupes (range: 4–20) were haphazardly collected underneath each individual. In total, 1639 fruits of 115 individuals were collected and then characterized in the laboratory. For each fruit, we visually estimated the percent of persisting pulp. After assessing the amount of persisting pulp, all drupes were carefully defleshed in order to search for boring insect holes in the endocarp.

### Statistical analysis

Data was analyzed using generalized linear mixed models using the SAS GLIMMIX macro [39]. This allowed modeling each response variable according to the particular distribution of their residues (e.g., binomial, Poisson). Target response variables were percentage of seeds predated and the average number of seed holes for each palm (considering only depredated seeds). The sample unit was always the individual plant and, thus, we estimated percentage of predated seeds and the mean number of holes per fruit for each sampled *C. humilis* in each population.

The explanatory variables considered were the geographic region (as defined above) and the type of population (human-altered or natural), and the percentage of persisting pulp (from 0–100%). In all analyses, population and plant (nested in population) were included as random factors. The adjusted means and standard errors were calculated using the LSMEANS option and were then back-transformed using the appropriate Taylor's series approach [39]. When the interaction between any two factors was significant, we performed tests for the effect of a given factor at the different levels of the other factor (“test of simple main effects”) using the Slice option in the LSMEANS [39].

## Results

### Frequency of seed predation

Mean percentages of *C. humilis* seed predation were highly variable among regions and also among localities in all four geographical regions (Fig. 1a). Seed predation was highest in Sevilla (56.2%±6.9; mean ±1ES), lowest in Cádiz-Málaga (25.8%±6.8), and intermediate in Valencia (37.8%±7.8) and Huelva (36.4%±4.2). Variation among populations within regions was also marked, ranging from 6.3% predation in Fuengirola to 66.1% predation in Reserva (Table 1). The mean percentage of persisting *C. humilis* fruit pulp also varied among populations, e.g. Saler (77.9%±7.2) *vs*. Alamillo (14.8%±5.1).

**Figure 2:**
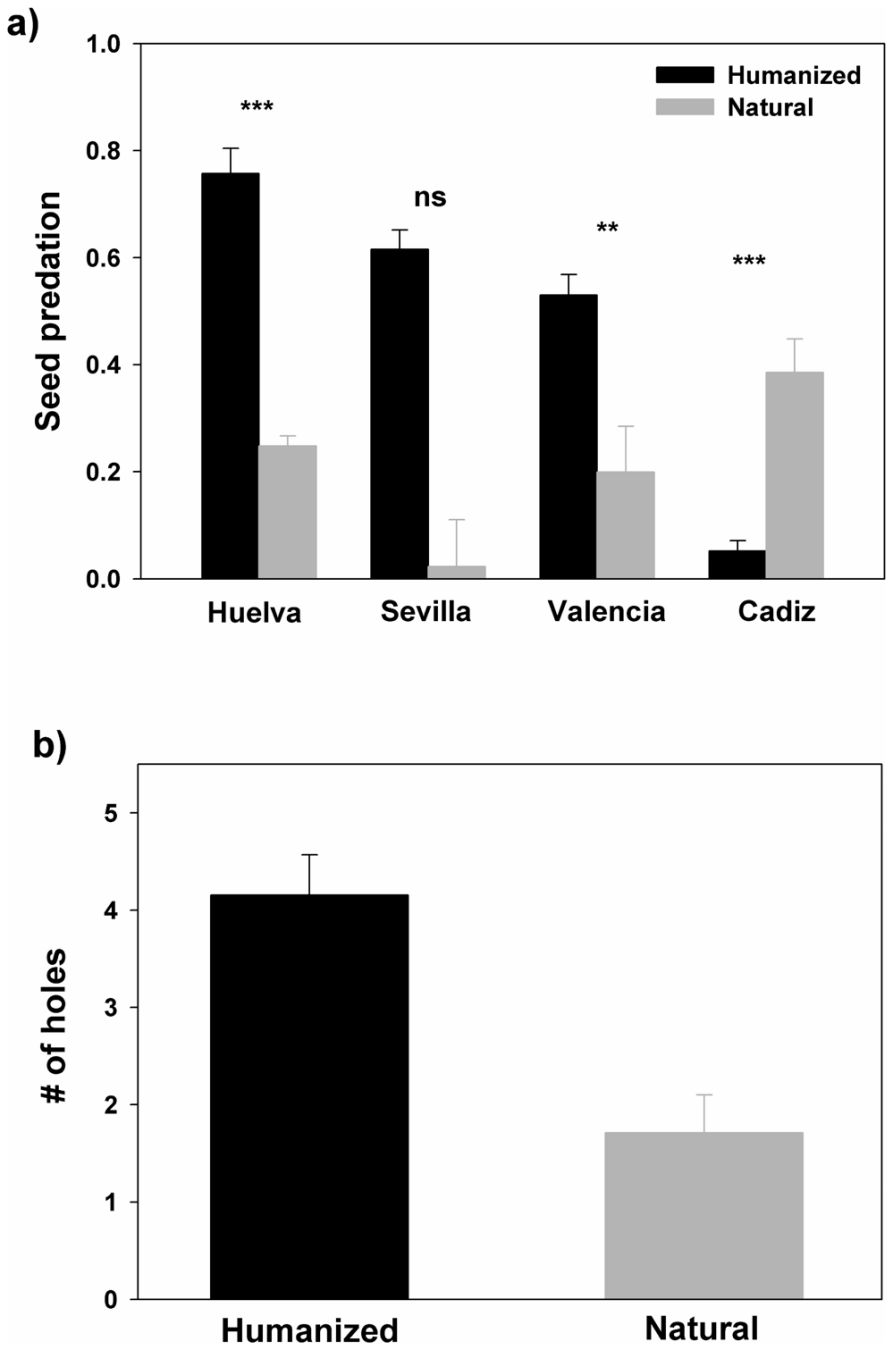
Factors explaining variation in *C. humilis* seed predation in the Iberian Peninsula. a) Model-adjusted mean percent seed predation in each geographical region for human-altered and natural populations. b) Model-adjusted mean number of beetle holes for human-altered and natural populations. Error bars represent standard errors.

**Figure 3:**
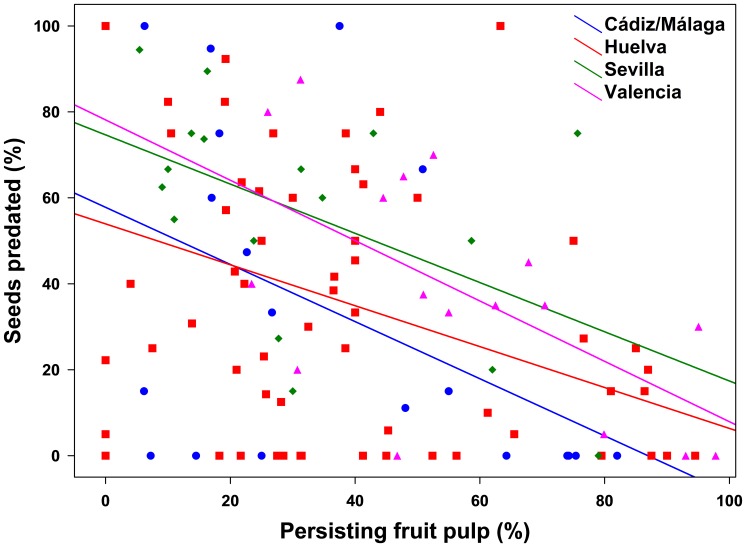
Negative linear relationships between percentage of seed predation and amount of persisting fruit pulp in each of the four target regions.

No significant effect was detected for the main factors in the GLM analysis (i.e., region, type of population, or percent of persisting fruit pulp; *P*> 0.249; Table 2). However, there was a strong significant interaction between population type and region (*P* <0.0001), indicating that the effect of population type (i.e. natural *vs*. human-altered) on seed predation was not consistent across regions. Specifically, tests of “slices” revealed that population type had a significant effect in Valencia (F_1,93_ = 7.32, *P*<0.009) and Huelva (F_1,93_ = 66.17, *P*<0.0001). As expected, the percentages of predation in human-altered populations of Valencia and Huelva were higher (3.1 and 2.7-fold, respectively) than in natural ones (Fig. 2a). Moreover, in Sevilla, there was also a much higher (over 27-fold) percentage of seed predation in human-altered than in natural populations (Fig. 2a). However, likely due to small sample size in natural populations, differences in this region were not significant (*P* = 0.286). In contrast, in Cádiz-Málaga we found on average a 9.2-fold higher percentage of seed predation in the natural population as compared with the human-altered population (Fig. 2a). Besides, there was a significant interaction between region and amount of persisting pulp (*P*<0.001), indicating that the effect of fruit pulp on seed predation was inconsistent across regions (Table 2). Indeed, though simple regressions showed that the relationship between amount of persisting pulp and percentage of seed predation was always negative and significant (r<−0.383, *P*<0.049, R^2^>0.209), the slope of the curve was higher in Cádiz/Málaga and Valencia as compared to Huelva and Sevilla (Fig. 3).

**Table 2:** Main results of our generalized mixed linear models testing the effect of region (Valencia, Sevilla, Huelva, Cádiz-Malaga,), type of population (human-altered, natural) and amount of persisting fruit pulp on *C. humilis* seed predation and the average number of invertebrate holes per preyed seed.

	Seed predation			Mean hole number		
	d.f.	F	*P*	d.f.	F	*P*
**Region (R)**	3, 6	0.54	0.670	3, 6	1.07	0.428
**Pulp (P)**	1, 93	1.34	0.249	1, 70	0.08	0.781
**Type (T)**	1, 93	0.91	0.342	1, 70	13.82	**0.0004**
R*T	3, 93	12.31	**<0.0001**	3, 70	1.66	0.183
P*R	3, 93	6.92	**0.0003**	3, 70	2.12	0.105
P*T	1, 93	1.24	0.268		-	
P*R*T	3, 93	2.10	0.106		-	

Significant differences (*P*<0.05) are marked in bold.

### Average number of holes per predated seed

The number of holes per seed predated was highly variable among localities in all four regions. The populations with the highest average number of holes per depredated seed were Viveros (6.1±0.9) and Alamillo (4.6±0.9) and the ones with lowest number of holes were Cantina (1.0±0.0) and Hinojos II (2.0±0.3).

Despite the fact the average number of holes per seed in human-altered populations (4.15±0.41) was about 2.4 times higher than in natural ones (1.71±0.39), a saturated model for such response did not revealed any significant effect likely due to its low statistical power. Indeed, after excluding the interaction between region and percentage of pulp from the saturated model, we found a significant effect (*P*<0.001) of population type on the average number of holes (Fig. 2b; Table 2). This trend was rather consistent across all regions, as indicated by the no detectable interaction between population type and region (*P* = 0.183; Table 2). Neither region (*P* = 0.428), nor percentage of persistent pulp (*P* = 0.781), nor interactions between factors had significant effects (Table 2).

## Discussion

Our study provides the first evidence indicating that *C. humilis* seed predation by insects and, in particular, introduced scolytine beetles, is widespread across the Iberian Peninsula, occurring in all studied regions and populations. These results are of interest not only because of the ecological and cultural importance of *C. humilis*, but also in the context of global change and on-going species invasions in the Mediterranean basin [2], [26].

The incidence of scolityne borers on palm crops is often high in both temperate and tropical species. Blumberg [33] quoted a 30–40% yield loss of unripe *P. dactylifera* dates attacked by *C. dactyliperda* in Israel, Zorzenon and Bergmann [40] noted about 80% reduction in germination of *Euterpe* spp. seeds attacked by *Xyleborus ferrugineous* in Brazil, and Janzen [14] found up to 99% of *Euterpe globosa* seeds damaged by *Coccotrypes carpophagus* in Puerto Rico. The only previous information about borer seed predation on wild *C. humilis* corresponds to a periurban population in Genova (Italy) and refers to a “massive infestation” by *D. longicollis* [37]. Such percentages of *C. humilis* seed predation are comparable to our estimates as approximately half of our sampled populations showed seed predation rates of 30–70%.

### Spatial variation in seed predation

Our results for *C. humilis* in the Iberian Peninsula support the worldwide pervasive trend of strong spatial variation in seed predation [7], [8], [41]. We found high variability in estimates of seed predation in most levels considered (i.e. among regions, within region, and within population). Variation among regions was not detectable presumably because of considerable variation among populations within a region (Fig. 1). For example, in Huelva, the percentage of seed predation at Chalet/Palacio was almost triple that found at Hinojos II (Table 1). Also, even within the same population, seed predation rate varied greatly among plants. For example, up to 95% of seeds of some plants were predated at Garganta Verde, while other plants did not have any predated seed. Also, we found similar spatial variation in the average number of entrance holes per predated seed at all scales considered (geographical, interpopulation, and within population; see Table 1). The myriad of biotic and abiotic factors potentially leading to such spatial variation in this plant-animal interaction certainly deserves further research [42].

### Effects of habitat degradation and amount of persisting pulp

Most identified borer beetles attacking *C. humilis* seeds belonged to two exotic species, *C. dactyliperda* and *D. longicollis*. These scolytine species probably were introduced in Spain with the date palm and the Canary palm [21], [23] and now attack other palm species [21]. Thus, it should be more probable to find infested *C. humilis* seeds in human-altered areas, where these palms are used commonly as ornamentals. As predicted, predation percentages in human-altered populations were much higher than in natural ones, except at Cádiz (Fig. 2b). The higher average number of entrance holes per seed in human-altered populations also support the idea that ornamental palms tended to enhance predation of *C. humilis* seeds by boring insects.

However, the effect of habitat degradation on seed predation was inconsistent across regions. Whereas human-altered populations of Huelva, Valencia and Sevilla had more infested seeds than natural populations, the trend was inversed in Cádiz-Málaga. The higher level of seed predation in the natural than human-altered populations of Cádiz-Málaga was unexpected and may relate to the fact we only sampled one natural and one human-altered population (Garganta Verde and Fuengirola, respectively). Thus, particularities of these two populations could have led to such an unexpected result. For instance, palm seeds infested by urban scolitid beetles are subjected to frequent management [43]. Thus, in some urban habitats fallen fruits (and infested seeds) are regularly removed by gardeners as yard waste, which could reduce some local beetle populations [43].

The fleshy pulp of many fruits acts as a chemical and/or physical defense against seed predators and pathogens [29], [44], including seed borer insects [14], [16]. Because the percentage of persisting *C. humilis* pulp was always negatively related to seed predation, our study supports the defensive hypothesis of fruit pulp [29], [44]. However, lack of pulp of sampled *C. humilis* fruits could relate to consumption by pulp feeders early after fruit fall [19] but also to pulp decay with fruit aging. In the later case, older defleshed seeds would have had longer time and thus higher chances of being attacked by seed predators. Though such a possibility cannot be ruled out, all evidence gathered during this and previous studies support the defensive role of *C. humilis* fruit pulp. For example, *C. dactyliperda* attacks on *C. humilis* (Authors *personal observation*) and date palms [33] fruits are sometimes unsuccessful because pulp secondary compounds deter (or even kill; [33]) these beetles. Also, *D. longicollis* attacks almost exclusively defleshed fruits or those with dry mesocarps [37]. Finally, Fedriani and Delibes [19] showed experimentally that fruits defleshed by seed dispersers experienced higher predation by borer insects (probably *D. longicollis* and other unidentified generalist seed predators) than whole intact fruits with pulp.

This is the first study on *C. humilis* seed predation across a geographical scale and reveals that introduced scolytines (and perhaps other beetles) are widespread seed predators, though their incidence was highly variable across the Iberian Peninsula. Seed predation strongly varied across a hierarchy of levels (among geographical regions, among populations, and within populations), with habitat degradation and the amount of defensive fleshy pulp influencing the level of seed predation. Interestingly, because human activity also can augment the population of some pulp consumers such as rabbits [45], it can lead to a number of synergic factors enhancing *C. humilis* proneness to seed predation. Further research concerning such possibilities as well as factors limiting *C. humilis* recruitment at different plant stages (seeds, seedlings, saplings) is clearly needed. This sort of study is particularly desirable given the charismatic nature of *C. humilis*, the high habitat degradation of the Mediterranean basin [46], and the current context of global change and on-going species invasions [2], [26].

## Supporting Information

Appendix S1 **This file contains data on percentage of** ***C. humilis*** **seed predation by beetles for each palm in each population.** Data on type of habitat (natural, humanized) and the mean percentage of persisting fruit pulp is also shown.
(XLSX)

Appendix S2 **This file contains data on the mean number of holes per seed for each** ***C. humilis*** **individual in each population.**
(XLSX)
